# Fiber-Based Thermoelectric Materials and Devices for Wearable Electronics

**DOI:** 10.3390/mi12080869

**Published:** 2021-07-24

**Authors:** Pengxiang Zhang, Biao Deng, Wenting Sun, Zijian Zheng, Weishu Liu

**Affiliations:** 1 Department of Materials Science and Engineering, Southern University and Science and Technology, Shenzhen 518055, China; 20039571r@connect.polyu.hk (P.Z.); 11849280@mail.sustech.edu.cn (B.D.); 11610114@mail.sustech.edu.cn (W.S.); 2 Institute of Textiles and Clothing, Research Institute of Intelligent Wearable System, The Hong Kong Polytechnic University, Hong Kong 999077, China; 3 Shenzhen Engineering Research Center for Novel Electronic Information Materials and Devices, Southern University of Science and Technology, Shenzhen 518055, China

**Keywords:** thermoelectric, fiber, wearable

## Abstract

Fiber-based thermoelectric materials and devices have the characteristics of light-weight, stability, and flexibility, which can be used in wearable electronics, attracting the wide attention of researchers. In this work, we present a review of state-of-the-art fiber-based thermoelectric material fabrication, device assembling, and its potential applications in temperature sensing, thermoelectric generation, and temperature management. In this mini review, we also shine some light on the potential application in the next generation of wearable electronics, and discuss the challenges and opportunities.

## 1. Introduction

In recent decades there has been a significant focus upon the combination of sensitive materials and flexible substrates with wearable electronics to realize functions such as sensing, health management, and environmental monitoring [1,2,3,4,5]. Owing to the increasing demand for power supply and people’s requirements for wearing comfort, flexible energy-harvesting devices with weight-light and self-powering have attracted increasing attention. Flexible generators that capture and convert ambient energy (solar, mechanical, thermal energy, etc.,) into electric energy are suitable candidates to achieve this goal because their flexibility and mechanical stability enable them to maintain their power generation function during deformation when they fit the human skin [6,7,8,9]. Among the above energy sources, thermal energy is a form of energy that can easily be reached and utilized. Life is maintained by the metabolism, resulting in a continuous heat dispersion from the human body with an energy density of 20 mW cm^−2^ [10]. Therefore, the human body is a sustainable heat resource for thermoelectric power generation.

Thermoelectric (TE) materials can directly realize the conversion between heat energy and electric energy base on the Seebeck effect and Peltier effect. Due to the exclusive advantages of solid-state conversion, long service life, and no moving parts, thermoelectric plays a vital role in solar energy collection, waste heat harvesting, and temperature management [11,12,13,14,15]. Thermoelectric technology also has excellent prospects for application in wearable electronics. However, the rigid architecture of conventional thermoelectric devices is not skin-conformal and comfortable for the real aim or wrist wearing [16]. Consequently, enormous efforts have been thrown into improving the flexibility of thermoelectric devices [17,18,19,20,21]. One strategy is to use flexible electrodes to replace the rigid electrode with a ceramic plate [20,22]. Another route is to make thermoelectric materials into thin films to increase their flexibility [18,23,24,25,26]. These devices have a certain degree of flexibility, but there are also many unsolved technique challenges. For example, flexible thermoelectric devices assembled with thermoelectric bulks have limited flexibility, restricting their ability to contact irregular surfaces. Thin-film flexible thermoelectric devices generally can be stretchable and bendable in one direction. Furthermore, since these devices lack air permeability, long-term wearing will make users feel uncomfortable.

**Figure 1:**
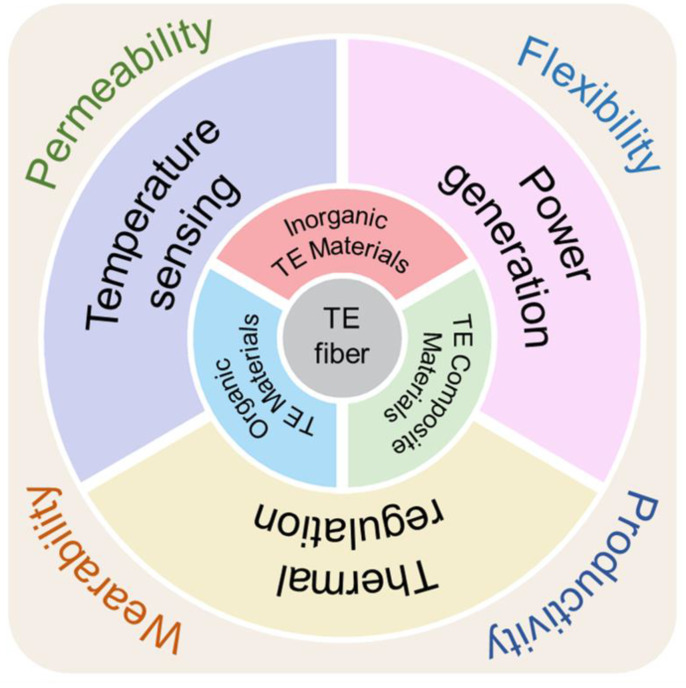
Fiber-based thermoelectric materials and devices for application in wearable electronics.

Fiber-based sensors and thermoelectric devices have gained wide attention in recent years due to their excellent mechanical properties and good wearing experience [27,28,29]. Figure 1 shows the current application scenarios and advantages of fiber-based thermoelectric materials and devices. It is very promising in wearable applications due to its excellent light-weight, flexibility, ductility, and in-plane shear properties. Compared with bulk-based flexible devices and thin-film flexible devices, fiber-based flexible devices have natural flexibility and good conformability in all directions. Consequently, fiber thermoelectric materials can act as functional components of thermoelectric devices and act as flexible substrates to improve the flexibility of devices. With very mature textile technology, it is possible to use fiber thermoelectric materials to fabricate large-scale wearable thermoelectric devices [30,31]. Considering the demanding potentials for fiber thermoelectric devices, we summarize the progress of fiber-based wearable thermoelectric materials and devices in recent years. Our scope covers fibrous materials, fabrication methods, and potential applications. Further, we give an outlook on the future directions, opportunities, and challenges of fiber thermoelectric devices with high performance. Together with some excellent review articles, we hope to draw more attention to fiber-based thermoelectric materials and devices [32,33,34].

## 2. Fabrication Methods for TE Fiber

**Figure 2:**
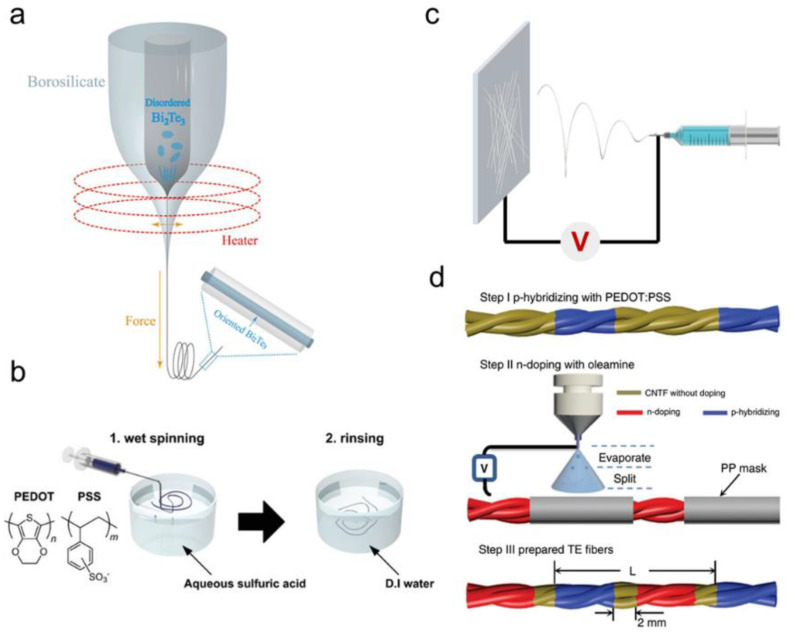
Fabrication methods for thermoelectric fibers. (**a**) Thermal co-drawing. Reproduced with permission from Ref. [41]. Copyright © 2018, AIP Publishing. (**b**) Wet spinning. Reproduced with permission from Ref. [42]. Copyright © 2020, Wiley-VCH. (**c**) Electrospinning. (**d**) Coating. Reproduced with permission from Ref. [43]. Copyright © 2020, Springer Nature.

Fabrication method, such as coatings, micro-nano fabrication techniques, fiber extrusion, thermal co-drawing, yarn formation, and fabric formation, are common methods for fabricating wearable electronic devices [35,36,37,38,39,40]. These methods can effectively transfer three-dimensional or two-dimensional functional materials into one-dimensional fibers, which dramatically improves the flexibility of available materials and expands the application of functional materials in the field of wearable electronics. Thermoelectric fibers are mainly prepared by thermal co-drawing, electrospinning, wet spinning, coating, and other methods (Figure 2).

### 2.1. Thermal Co-Drawing

Thermal co-drawing is a method for preparing inorganic fibers and has been widely used to produce fibers with uniform longitudinal direction [41,44,45]. The inorganic core material is wrapped in a glassy material to prepare a preform, which is placed in a heater to make it soft. Then the blank is pulled at a suitable speed to form a viscous flow, which can finally be elongated into fibers with micron-scale diameters. Yang et al. prepared a Sn-Se alloy core borosilicate glass-clad fiber with a core diameter of 94 µm by thermal co-drawing [46]. The TE fiber core was composed of SnSe polycrystalline grains and had a high Seebeck coefficient of −151 µV K^−1^ at 300 K. They used the same method to fabricate bismuth selenide fibers with a diameter of 50 μm [47]. Because low-dimensional materials can improve thermoelectric performance [48], the Seebeck coefficient of the Bi_2_Se_3_ fiber can be up to −150 µV K^−1^, much higher than that of bulk Bi_2_Se_3_. Zhang et al. prepared single-crystalline SnSe fibers by thermal co-drawing and recrystallization process by using a CO_2_ laser [49]. The experimental results showed that the single-crystal SnSe fiber had good thermoelectric properties, and the ZT value can reach 2 at 862 K.

### 2.2. Electrospinning

Electrospinning is a commonly used dry spinning process, producing polymer fibers with a submicron-scale diameter. Before the electrospinning process, the polymer and the solvent are processed into a high-viscosity polymer solution. Under the high-voltage electric field, the charged polymer jet passing through the needle tip is gradually concentrated and solidified into fibers. Maensiri and Nuansing demonstrated the preparation of thermoelectric oxide NaCo_2_O_4_ nanofibers by electrospinning for the first time [50]. First, sodium acetate, cobalt acetate, and polyacrylonitrile were fully stirred in a solvent to form a homogeneous polymer solution, and then the fiber precursor was formed at a voltage of 20 kV. Finally, sodium cobalt oxide nanofibers with a diameter of 20~200 nm were prepared by calcination. Kim et al. prepared ZnO/polyvinylpyrrolidone composite nanofibers using zinc acetate dihydrate, polyvinylpyrrolidone, and ethanol as precursor solutions for electrospinning at a voltage of 8 kV [51]. After rapid annealing, the mesoscale grains of the composite nanofibers scatter phonons and electrons more obviously, and the Seebeck coefficient at room temperature was 313.7 μV K^−1^, which was about 3.8 times that of the composite nanofibers without a grain structure.

### 2.3. Wet Spinning

Wet spinning is another standard method for preparing polymer fibers. The conductive polymer and solvent are mixed to obtain a spinnable stock solution. Then the spinning stock solution is ejected at an appropriate speed through a spinneret immersed in a coagulation bath to precipitate and solidify the fibers. Jang et al. used a ball mill to mix poly (3,4-ethylenedioxythiophene) polystyrene sulfonate (PEDOT: PSS) spheres, carbon nanotubes, and deionized water into spinning stock solution and then extruded it into the coagulation bath of methanol at a flow rate of 4 mL/min to prepare carbon nanotubes (CNT)/PEDOT: PSS composite fiber [52]. After post-treatment, the thermoelectric properties of CNT/PEDOT: PSS composite fibers were optimized, and the power factors of P-type and N-type were 83.2 ± 6.4 and 113 ± 25 µW m^−1^ K^−2^, respectively. Jang et al. also investigated the effect of single-walled carbon nanotubes (SWCNT) content and dispersion on the properties of thermoelectric fibers by using insulating polymer poly(vinylidene fluoride) (PVDF) as the polymer matrix for wet spinning [53]. The results showed that the electrical conductivity of the optimized SWCNT/PVDF composite fiber was as high as 1950 ± 483 S cm^−1^, and the P-type and N-type power factors of the optimized SWCNT/PVDF composite fiber were the highest among all the reported data.

### 2.4. Coating

Fabrics, yarns, and threads can be converted into wearable, biologically compatible thermoelectric devices upon being coated with films of thermoelectric materials. Coatings can be prepared on traditional textile materials by a variety of methods, including dip coating, vapor deposition, electrochemical deposition, in situ solution polymerization, and inkjet printing [54]. These techniques can be extended to the textile industry on a large scale with cost advantages. Jiang et al. used the dip-coating method to soak ordinary cotton fabric in PEDOT: PSS solution containing 5 vol. % dimethyl sulfoxide and then repeated the above process after taking it out and drying to absorb different amounts of PEDOT: PSS on the surface of cotton fabric, which had a relatively high conductivity of 18.8 S cm^−1^ [55]. Snyder et al. prepared P-N alternate thermoelectric fibers on carbon nanotube fibers using a coating technique [43]. First, carbon nanotube fibers were dipped into a commercial PEDOT: PSS solution for P-hybridization. Then, N-type carbon nanotube fibers were obtained at equal intervals by using polypropylene as a mask and oleamide doping combined with an electric spraying technique. Finally, thermoelectric fibers with alternately doped N or P segments were formed.

## 3. Thermoelectric Fiber Materials

The choice of appropriate materials is of most importance for the design and construction of fiber-based thermoelectric devices, which require the materials to have the features of high thermoelectric figure-of-merit, excellent mechanical properties, eco-friendliness, and low cost. The following section presents the latest progress in fiber-based inorganic thermoelectric materials, organic thermoelectric materials, and composite thermoelectric materials. Selective progress in fiber-based thermoelectric materials are summarized in Table 1.

### 3.1. Inorganic TE Fibers

Since discovering the thermoelectric effect, inorganic thermoelectric materials have been widely investigated [64,65,66,67]. Although many inorganic materials have a high ZT at high temperatures, the working temperature of wearable thermoelectric devices is around 310 K, limiting the application of inorganic thermoelectric materials in the field of wearable electronics. Bi_2_Te_3_ is a classic room-temperature thermoelectric material, which has the characteristics of good room temperature stability, high electrical conductivity, and low thermal conductivity, and has been rapidly developed in recent years [68,69,70]. In 2001, Stacy et al. prepared dense and continuous Bi_2_Te_3_ wires with a diameter of 40 nm in a porous alumina template by the electrodeposition method [71]. The deposited wire had a high texture in the (110) direction. Subsequently, Kaviany et al. studied phonon and electron transport in Bi_2_Te_3_ using a multi-scale approach, demonstrating the potential of low-dimensional Bi_2_Te_3_ materials for wearable thermoelectric devices [72]. Yang et al. prepared Bi_2_Te_3_ glass-clad thermoelectric fiber by thermal co-drawing [41]. The fiber core was composed of polycrystalline hexagonal nanosheets with a preferred orientation. The ZT value of the fiber at 300 K was as high as 0.73. However, due to the inherent brittleness of inorganic materials, their application in flexible electronics is greatly limited. Recently, Mg_3+δ_Sb_1-x_Bi_x_ family has emerged as a new promising room-temperature thermoelectric with high toughness, which could be a better candidate for the fibers [73,74,75,76,77].

### 3.2. Organic TE Fibers

The organic conductive polymers such as polyaniline (PANI), polypyrrole (PPy), polythiophenes (PTs), PEDOT: PSS, and polyacetylene (PA) have inherent flexibility and good adhesion to the skin, so they can make better use of the temperature difference between the human body and the environment [78,79,80]. In contrast to traditional inorganic materials, organic conductive polymers have the characteristics of low thermal conductivity, light-weight, softness, low toxicity, and convenient production. It is believed that organic TE materials are better for next-generation wearable thermoelectric devices. PEDOT:PSS is considered to be one of the most promising conductive polymers for wearable thermoelectric devices. In recent years, the thermoelectric properties of PEDOT:PSS fibers have been extensively studied, and some remarkable progress has been made [81,82]. Xu et al. explored the influence of different solvents on the properties of PEDOT:PSS fiber, showing a significant impact on the gelation process of PEDOT:PSS [57]. While the post-treatment with organic solvents of ethylene glycol and dimethyl sulphoxide significantly increased the electrical conductivity up to two times. Weisenberger et al. developed a continuous wet-spinning process to prepare PEDOT:PSS fibers with high electrical conductivity, excellent mechanical properties, and moderate thermoelectric properties [83]. They found that stretching the fiber induced a preferred orientation of the polymer chains along the fiber axial direction, resulting in superior electrical, thermal, and mechanical properties. The conductivity was saturated at about 2000 S cm^−1^ at room temperature with increasing tensile strength, while the Seebeck coefficient was almost constant with stretch. Although organic thermoelectric materials have many benefits, their low Seebeck coefficient and low conductivity limit their large-scale application in the wearable field. Moreover, due to the relatively complex system of organic thermoelectric materials and lack of systematic investigation, new strategies are needed to design organic thermoelectric materials and fibers.

### 3.3. TE Composite Fibers

Thermoelectric organic/inorganic composite material usually has a balance between the flexibility of organic materials and the high thermoelectric ZT of inorganic materials, and hence is widely used for TE fibers [84,85,86]. Segalman et al. have fabricated a PEDOT: PSS/Te-nanowire composite fibers, which has an optimized power factor of 100 µW m^−1^ K^−2^, which was nearly five orders of magnitude higher than that of pure PEDOT:PSS [87]. Wu et al. used a coating method to prepare p-type and n-type Ag_2_Te nanocrystals on the surface of nylon fibers to form a flexible composite thermoelectric material [88]. At the temperature difference of 20 °C, a thermoelectric generator prepared with such fiber can generate about 0.8 nW of power. Carbon-based thermoelectric composite fiber was another promising choice. Zhu et al. prepared high-performance thermoelectric composites using carbon fiber as filler and poly (3-alkylthiophene) (P3AT) as matrix [60]. The carbon fiber provided a good conductive path in the composites, and the optimized composites had of electrical conductivity of 380 S m^−1^ and Seebeck coefficient of 136 μVk^−1^. Through various optimization methods to improve the chemical incompatibility between organic materials and inorganic materials, the thermoelectric properties of composite materials have been greatly improved. However, a significant gap still exists as compared to the inorganic bulk materials. Therefore, the development of high-performance composite thermoelectric materials has become the top priority for producing stable and reliable wearable thermoelectric fabrics.

## 4. Fiber-Based Thermoelectric Devices and Applications

Thermoelectric fibers can adapt to complex deformation through stretching or twisting, which can be woven or knitted into functional textiles with higher dimensional structures in various configurations and integrated into a large-area array. Thermoelectric fabrics have good flexibility, good stretchability, and air permeability, and some are even washable, which can significantly expand the application of thermoelectric fabrics in wearable electronics. This section will go through the research progress of fiber-based wearable thermoelectric devices, including temperature sensors, thermoelectric generators, and temperature controllers.

### 4.1. Temperature Sensing

Temperature is one of the most important physical quantities for the human body to perceive the environment, as well as an essential health indicator that responds to physiological activity. Since the COVID-19 pandemic, body temperature measurement has become an essential part of keeping the public safe, usually using thermometers and thermal imaging. It is helpful to realize intelligent healthcare by using wearable electronic devices to monitor the temperature data of the environment or human body in real-time. TE fibers are highly flexible and can be woven or knitted into a variety of functional textiles, such as masks and clothing, making them a feasible material for the development of health information collection and tracking devices. The thermocouple is a temperature sensor based on the Seebeck effect for temperature measurement. The thermocouple involves connecting two different metals or semiconductors with a voltage meter to form a closed-circuit loop. When the temperature of the two junctions is different, an electromotive force is generated in the loop. With the advantages of small size, fast response speed, low cost, and wide working range, a flexible thermocouple is an ideal component for the scenario of the Internet of Things in the future. Due to the inherent flexibility and two-dimensional network structure, TE fiber-derived thermocouple sensors possess better mechanical flexibility and conformability to be well attached to complex surfaces for accurate temperature measurements.

**Figure 3:**
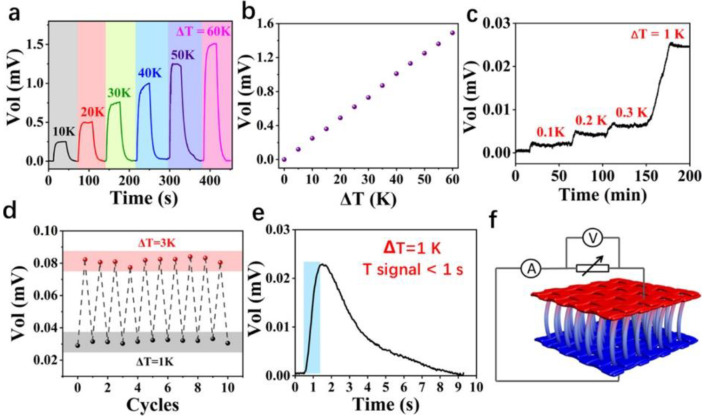
Temperature-sensing performance of fiber-based thermoelectric devices. Reproduced with permission from Ref. [30]. Copyright © 2020, American Chemical Society. (**a**) The relationship between the output voltage and ΔT. (**b**) The Seebeck coefficient of the thermoelectric device is simulated from the Voltage-ΔT curve. (**c**) The temperature response resolution is as small as 0.1 °C. (**d**) The temperature sensor is a stable temperature response. (**e**) The response time of temperature sensing. (**f**) The temperature-sensing mechanism.

Wei et al. prepared flexible thermoelectric fibers by thermal co-drawing a semiconducting glass as the core and a thermoplastic polymer as the cladding [89]. The TE fiber showed good mechanical flexibility and excellent temperature-sensing performance. The temperature measurement range could be up to 150 °C with a resolution higher than 0.05 °C, suggesting a promising temperature sensor. Wei et al. also sewed the fiber into the textile to form a 3 × 3 temperature-sensing array, locating heat sources at millimeter resolution while achieving high spatial resolution thermal sensing and positioning. Wang et al. used modified PEDOT:PSS to assemble a self-powered pressure–temperature-sensing electronic device on a 3D spacer fabric [30], as shown in Figure 3. The smart fabric had a Seebeck coefficient of 25 µV K^−1^, which can efficiently and accurately detect temperature with a detection resolution of 0.1 °C and a response time of 1 s. A flat temperature measurement array can be realized through the cross arrangement of electrodes, which can provide 2D-temperature mapping, and made it possible to prepare a large-area wearable smart fabric that measures the temperature distribution.

Thermoelectric fiber-based temperature sensors have shown their potential in the field of wearable electronics. However, the sensitivity of the temperature sensor strongly depends on the voltage meters. Increasing the Seebeck coefficient of the TE fibers, or using a multi-connected thermocouple is a direction

### 4.2. Power Generation

Thermoelectric generation (TEG) technology converts thermal energy into electrical energy by contacting objects with temperature differences. The fiber-based thermoelectric devices have the advantages of being flexible and wearable, which is a good candidate for wearable electronics’ energy supply. Textiles have become ideal tools for carrying energy conversion equipment since clothing is a necessity for human beings. Embedding thermoelectric fibers in fabrics to manufacture fiber-based thermoelectric devices is an effective method for self-powering wearable electronics. The knitting, embroidery, and other textile processes were used to assemble the flexible thermoelectric generators [33]. In a real wearable scenario, the skin is usually used as the hot side while air is the cold side, which was the most effective way to utilize the body–environment temperature gradient while keeping our body comfortable.

**Figure 4:**
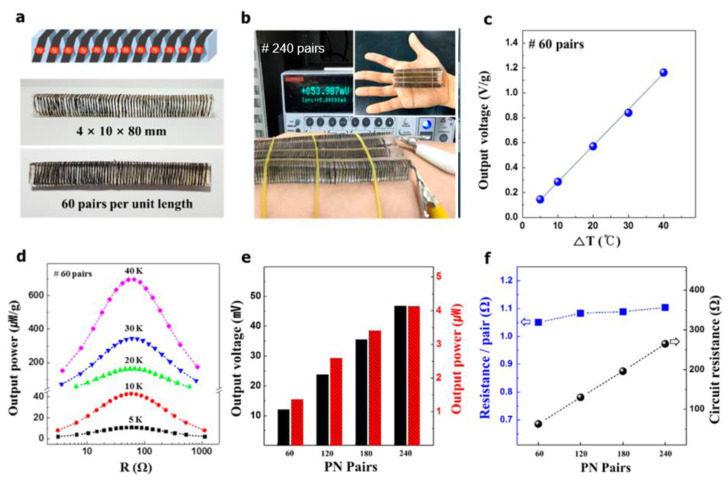
Performance of fiber-based thermoelectric generators. Reproduced with permission from Ref. [90]. Copyright © 2017, American Chemical Society. (**a**) Photograph of a thermoelectric generator with 60 P-N pairs. (**b**) The output voltage of the thermoelectric generator at room temperature. (**c**) The relationship between the output voltage density of the thermoelectric generator and the temperature difference. (**d**) The relationship between the output power density of the thermoelectric generator and the load resistance. (**e**) The output voltage and power of thermoelectric generators with different numbers of P-N pairs at ΔT = 5 °C. (**f**) The average resistance of each P-N pair and the circuit resistance of the thermoelectric generator at ΔT = 5 °C.

Li et al. prepared a fiber-based thermoelectric generator by depositing synthetic Bi_2_Te_3_ and Sb_2_Te_3_ particles into a commercial silk fabric [17]. The generator can effectively convert thermal energy into electrical energy within the temperature range of 5~35 °C, with a maximum output voltage of 10 mV and output power of 15 nW. They also studied the effect of wearing it on the arm, which produced a voltage output of 6.02 mV. Kim et al. alternately doped the synthesized high-performance carbon nanotube yarn with polyethyleneimine and FeCl_3_ into n-type and p-type, and then wound it on a polydimethylsiloxane support unit to form a wearable thermoelectric generator, as shown in Figure 4 [90]. Under the temperature difference of 5 °C, the authors obtained a maximum power density of 0.41 μW cm^-2^ in the flexible thermoelectric generator based on 60 P-N pairs. In practical application, when a flexible thermoelectric generator with 240 P-N pairs was placed on a human arm, the temperature difference between the hot side and the cold side reached 6 °C, and the output voltage was about 54 mV. In addition, many wearable thermoelectric fabrics also exhibit good power generation capabilities under idea temperature differences [91,92,93].

Fiber-based thermoelectric devices have shown many advantages in the conversion of human heat. However, the output performance of these devices is still low due to the small temperature difference between the human body and the environment. Future research should focus on improving the properties of thermoelectric materials and building stable temperature differences to enhance power generation capacity.

### 4.3. Thermal Regulation

Temperature regulation is essential to improve the thermal comfort of the human body for the application of smart fabrics. The heat exchange methods between human skin and the environment mainly include conduction, radiation, evaporation, and convection. By influencing these heat exchange modes, people have developed various smart fabrics for temperature regulation, including electrothermal conversion, phase change heat storage, radiant cooling, and so on [94,95,96,97,98,99]. These fabrics exhibit excellent temperature regulation capabilities in specific environments. However, most of them only have a one-way adjustment function, which cannot maintain the thermal comfort of the human body in a complex environment. Thermoelectric materials can actively control the temperature, which is suitable for personalized temperature control. Integrating thermoelectric equipment into clothing systems for personalized temperature regulation is a promising solution to energy waste and climate change. When the human body is in a high-temperature environment, the cold side of the thermoelectric device is attached to the skin surface for cooling, so that the human body can feel the cold. When the human body is in a low-temperature environment, the hot side of the thermoelectric device is attached to the skin surface for heating, so that the human body can feel the warmth. This is an active form of temperature regulation that can be used to heat or cool objects. By adjusting the size and direction of the current, thermoelectric devices can keep the body feel comfortable in complex temperature environments. Although thermoelectric fabrics have great potential in regulating human body temperature, the current thermoelectric temperature regulation devices are mostly made of bulk materials and flexible substrates because of the poor thermoelectric properties of fiber-based thermoelectric materials.

**Figure 5:**
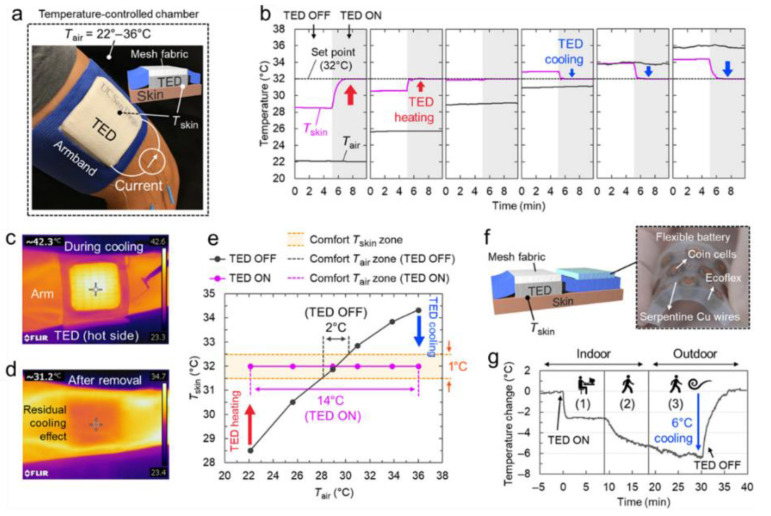
The performance of thermoelectric temperature control devices. Reproduced with permission from Ref. [100]. Copyright © 2019, AAAS. (**a**) Photo and schematic diagram of thermoelectric temperature control fabric. (**b**) The skin temperature changes before and after the thermoelectric control fabric works under different air conditions. (**c**) The infrared image of the thermoelectrically controlled fabric (hot side) when the subject’s arm is cooling (I = 160 mA). (**d**) Remove the infrared image of the thermoelectrically regulated fabric skin, showing the residual cooling effect. (**e**) The temperature of the skin surface changes with and without thermoelectric control fabric operation. (**f**) Schematic diagram and photo of the thermoelectric control fabric integrated with the flexible battery pack. (**g**) Under different conditions, the skin temperature changes caused by the cooling of the fabric through thermoelectric regulation.

Chen et al. demonstrated a flexible thermoelectric device with active temperature regulation capability, as shown in Figure 5 [100]. In this design, rigid inorganic thermoelectric legs were embedded in two layers of stretchable substrates. The air gap between the substrates greatly reduced the thermal conductivity between the hot and cold sides of the thermoelectric devices, thus providing an excellent temperature regulation effect. The device was integrated into the clothing and worn on the human arm in the actual wearing situation. The fabric system used proportional-integral-derivative (PID) to control the size and direction of the current. When the ambient temperature was high (namely, 22°, 26°, 29° and 31 °C), the thermoelectric device cooled the skin surface. When the ambient temperature was low (namely, 34° and 36 °C), the thermoelectric device heated the skin surface, ensuring that the skin surface temperature of the human body was around 32 °C to make a comfortable feeling.

Although various wearable thermoelectric generators can be used for cooling in principle, the requirements to achieve active cooling are much stricter, so the current research progress on thermoelectric fabrics for temperature management is slow. One of the main disadvantages of the fiber TEG is the high joule heat when used for cooling due to the high resistance compared with its bulk counterpart. In addition, heat dissipation at the hot side of a thermoelectric device is also a challenge because all the fabric materials, human skin, and air are poor conductors of heat, and fabrics have a large thermal resistance. The external thermal resistance significantly reduces the device’s intrinsic thermoelectric performance, which presents a fundamental challenge to achieve efficient physical applications.

## 5. Conclusions and Outlook

Fiber-based thermoelectric materials and devices show great potential in wearable electronic devices due to their excellent flexibility, excellent air permeability, and considerable energy conversion performance. We reviewed the development of fiber thermoelectric materials and devices in recent years from the materials, preparation methods, and device applications. Generally, inorganic thermoelectric fibers have good thermoelectric properties, but the poor flexibility and complex processing technology limit their application in wearable thermoelectric devices. Organic thermoelectric fibers have good flexibility, but their thermoelectric properties need to be further improved. It is still a problem for hybrid fibers to improve their thermoelectric properties without compromising their flexibility. Thermoelectric devices made from thermoelectric fibers have three main applications: temperature sensing, thermoelectric generation, and temperature management. Because of its strong deformability, thermoelectric fibers can be woven into the fabric for temperature sensing. Thermoelectric generators integrated with thermoelectric fiber have also attracted extensive attention due to their excellent wearable performance and their ability to use body temperature for the self-power supply of wearable devices. The thermoelectric temperature regulation fabrics based on the Peltier effect have sufficient development space to realize personalized temperature management.

Although fiber thermoelectric materials and devices have great application potential, they are still in their infancy and far from commercial production. Several key questions about materials and devices need to be addressed to facilitate the rapid development of fiber-based thermoelectric materials and devices in wearable electronics. (1) Improving the thermoelectric properties and stability of fiber materials. Both organic and inorganic materials have a trade-off between flexibility and thermoelectric properties. It is necessary to explore the fundamental decoupling mechanism relative to the thermoelectric fibers or to discover new materials with intrinsic high TE properties and excellent flexibility. Moreover, environmental stability, especially humidity stability, needs more attention, as the resistance of fiber thermoelectric materials made of water-soluble polymers such as PEDOT:PSS is affected by humidity and thus leads to cross-talk with temperature [101]. (2) Optimizing the structure of fiber-based thermoelectric devices. As an object that contacts human skin, the structure of fabric dramatically affects the performance of thermoelectric devices. It is highly beneficial to reduce the resistance and contact thermal resistance and maximize the temperature difference between the human body and the environment through proper braiding technology to efficiently and flexibly arrange the p-type structure and n-type structure of thermoelectric devices. One possible approach is to use structures beyond the plane, such as whisker arrays, for more sensitive temperature sensing. (3) Standardizing test methods and evaluation criteria. Currently, it still lacks standardized measurement and characterization methods. Therefore, a generally recognized criterion is needed to measure the performance of wearable devices under actual application conditions.

It is also worth noting that the body’s perception of thermal comfort in the environment is affected by different modes of heat exchange. However, conventional temperature sensors can only simply perform temperature measurement but not a quantitative characterization of thermal comfort. TE fibers, due to their cross-linked structure, are promising to achieve multimodal sensings such as pressure sensing, humidity sensing, and light-sensing based on temperature sensing by blending different materials, thus characterizing and regulating human thermal comfort more realistically, showing a promising application in the field of wearable electronics in the future. 

## Figures and Tables

**Table 1:** Summary of some popular thermoelectric fiber materials and their performance.

Materials	$S\left(\mu V\cdot K^{-1}\right)$	$\sigma \left(S\cdot cm^{-1}\right)$	$\kappa \left(W\cdot m^{-1}\cdot K^{-1}\right)$	ZT	T(K)	Ref.
Inorganic fibers						
Bi_2_Te_3_	130.5	744	0.52	0.73	300	[41]
Bi_2_Se_3_	−150.85	319	1.25	0.18	300	[47]
SnSe	306.9	56.4	0.25	2	862	[49]
Sb_2_Te_3_	−176	88	1.2	0.07	-	[29]
Organic fibers						
PEDOT:PSS	72	950	0.42	0.42	297	[56]
PEDOT:PSS	14.8	172.5	-	-	-	[57]
Poly (3-hexylthiophene)	14.8	50	0.0708	0.016	-	[58]
PEDOT	23	869	0.37	0.036	-	[59]
Composite fibers						
Poly (3-octylthiophene)-CNT	136	3.6	-	-	-	[60]
PEDOT:PSS-Te	115	215	0.2	0.39	-	[61]
PEDOT:PSS-CNT	70.1	1043.5	0.4–1.0	-	-	[62]
Cellulose- Bi_2_Te_3_	−134.2	209.6	0.47	0.38	437	[63]
